# A Sparse Representation-Based Deployment Method for Optimizing the Observation Quality of Camera Networks

**DOI:** 10.3390/s130911453

**Published:** 2013-08-28

**Authors:** Chang Wang, Fei Qi, Guangming Shi, Xiaotian Wang

**Affiliations:** School of Electronic Engineering, Xidian University, Xi’an, Shaanxi, 710071, China; E-Mails: gmshi@xidian.edu.cn (G.S.); xtwang@mail.xidian.edu.cn (X.W.)

**Keywords:** camera networks, deployment, nodes layout and assignment, sparse representation, anisotropic sensing model, observation quality

## Abstract

Deployment is a critical issue affecting the quality of service of camera networks. The deployment aims at adopting the least number of cameras to cover the whole scene, which may have obstacles to occlude the line of sight, with expected observation quality. This is generally formulated as a non-convex optimization problem, which is hard to solve in polynomial time. In this paper, we propose an efficient convex solution for deployment optimizing the observation quality based on a novel anisotropic sensing model of cameras, which provides a reliable measurement of the observation quality. The deployment is formulated as the selection of a subset of nodes from a redundant initial deployment with numerous cameras, which is an *ℓ*_0_ minimization problem. Then, we relax this non-convex optimization to a convex *ℓ*_1_ minimization employing the sparse representation. Therefore, the high quality deployment is efficiently obtained via convex optimization. Simulation results confirm the effectiveness of the proposed camera deployment algorithms.

## 1. Introduction

Camera networks, as a special category of senor networks with all nodes being cameras, collect comprehensive and reliable visual information of regions being monitored based on well-planned deployment of cameras in the scene. It is widely applied in traffic management [1], security monitoring [2], agricultural production [3], virtual reality [4], intelligent surveillance [5], stereo reconstruction [6], *etc.* The node deployment, which aims at optimizing both the quality of captured information and the cost of visual information acquisition, is an essential step in camera network related applications [7].

The camera deployment contains two aspects, which are the static layout and the dynamic assignment. Layout and assignment approaches are designed to optimize the distribution of observation quality and the resource consumption in different stages of camera deployment. The task of static layout is to efficiently place the least number of cameras with well-designed positions and parameters, such as the orientation, to achieve an optimized observation quality distribution over the whole scene. Once a layout has been determined, the dynamic assignment mechanism controls the working states of cameras in real time to operate the network with the least number of active cameras to reduce the resource consumption. In addition, the sensing model of cameras, which can provide a realistic measurement of observation quality, is also of importance in the layout and assignment.

### 1.1. Deployment

Effective and efficient approaches for the deployment of camera networks are expected for real-world applications. An empirical manual deployment is efficient and practical for small scenes. However, this approach makes no assurances on the coverage, observation quality and hardware cost. Thus, it is hard to extend to complex and large scale scenes. To deploy camera networks, both layout and assignment should be considered to optimize the overall observation quality under constraints, including the number of cameras, energy supply, communication bandwidth, *etc.* As it requires selection and adjustment on the configurations of cameras, such as positions and orientations, the deployment is intrinsically a combinatorial optimization problem, which faces the well-known NP-hard problems [8]. Furthermore, the overall measurement of observation quality over a scene is non-convex, even if it is quasi-convex for one camera. This makes the deployment a non-convex optimization problem, which is hard to be solved efficiently.

Extensive works on deployment have been done for various sensor networks. In general sensor networks, researchers mainly focus on omni-directional sensors and deploying cameras according to the energy consumption, coverage and connectivity of communication nodes [9–11]. For camera networks, various deployment schemes have been investigated. Approaches employing integer programming [8,12] are proposed for optimizing the coverage based on the binary sensing model. Sensors with the binary sensing model can sense the object in its field of view with a quality of one; and the sensing quality out of its field of view is zero. To reduce the energy and bandwidth consumption in resource-constrained networks, sensor deployment strategies are designed in [13–15]. This literature considers only the coverage of the observed fields, which cannot comprehensively reflect the observation quality. In addition, the concept of multiple level coverage, or *K*-coverage, for short, is introduced in [16] to assure that one object is detected by at least *K* sensors. This is of importance for binary sensing models. However, the *K*-coverage model makes the problem more complex, as the potential combinations of positions and poses of cameras are increased exponentially.

Facing the high difficulty and computational complexity of sensor deployment, several algorithms, such as integer programming [8,12], particle swarm optimization [17,18], genetic programming [19,20] and the bee colony algorithm [21], have been introduced for finding the solutions. However, these approaches can hardly achieve high efficiency, because the related problems they deal with are still non-convex.

According to the analysis above, for the camera network, it is hard to obtain an effective and efficient solution of deployment, due to its combinatorial nature. Fortunately, the recent development in compressed sensing [22] shows that the combinatorial *ℓ*_0_ pseudo-norm minimization can be relaxed to a convex *ℓ*_1_ optimization with an exact solution, supposing the vector (signal) is sufficiently sparse [23,24]. Furthermore, the theory also shows that the sparsity is a widely existing nature of signals. Thus, we can apply such a convex relaxation if we can construct (or find) a sparse representation of the problem. In fact, in sensor networks, the sparse representation has been applied in multiple source data fusion and analysis [25]. Next, we need to construct a sparse representation suitable for the deployment problem.

Different from the existing approaches, which obtain the solution through computational intelligent or integer programming, our approach solves the problem via convex relaxation, so that the deployment can be solved efficiently. In this paper, the deployment is solved by selecting the best subset of cameras from a redundant initial layout. As the number of cameras in the initial layout is very large compared to the number of selected ones, the vector indicating which camera is selected is sparse. Such a formulation is based on the fact that the redundant layout includes the near-optimal deployment. If we randomly place a number of cameras in the scene as an initial layout, a sub-optimal deployment can be obtained by selecting a subset from this layout. The sub-optimal deployment is near-optimal with satisfactory observation quality if the number of cameras in the initial layout is large enough. According to the research on coverage [26], *O*(*A*/*R*^2^) cameras with omni-binary sensing fields guarantee a full coverage of a field of area, *A*, where *R* is the radius of the sensing range. In the initial layout, if it is ten or more times larger than this threshold, the number of cameras is large enough to obtain a near-optimal deployment. In addition, for a sensing model with a non-circular coverage shape, we can use the radius of its maximum inner circle instead to get an over-estimated coverage threshold.

Based on the sparse representation, we propose a framework to deploy camera networks by minimizing the pseudo *ℓ*_0_ norm of an indicator vector, given constraints on observation quality. A systematic approach is provided to get the representation and compute the observation quality.

### 1.2. The Sensing Model

The camera, as a special kind of sensor, has attracted much interest in the measurement of its observation quality [27]. In the art gallery problem [28], a view point watches any position with equal quality, as long as there are no obstacles between them. Considering the limited sensing field angle, a directional binary sensing model is designed in [8]. Two non-uniform directional models are proposed in [17,20,29] under the consideration of anisotropic sensing characteristics. However, both models are based on empirical assumptions, which do NOT provide realistic measurements of observation quality. Thus, a measurement based on the physical imaging process is expected.

In this paper, we consider the camera networks composed of cameras with directional, occlusive and nonuniform sensing fields. We measure the observation quality of a given position in the scene by the quality of its image. In this work, we consider a widely used camera with such a structure, which is equipped with a lens with a fixed focal length and without auto-focus capability. To construct its sensing model, we explore the relationship between the imaging quality and four relevant factors: resolution, defocus, geometric distortion and occlusion. All factors are crucial to feature extraction and image analysis and related directly to the configuration of a camera. For each factor, a measurement function is designed according to its influence on the imaging quality. Thus, the anisotropic sensing model for cameras is derived by combining the four parts.

In this paper, an efficient approach is proposed to deploy camera networks for large-scale complex scenes, which solves the original non-convex NP-hard combinatorial optimization problem via convex relaxation by introducing the sparse representation idea. The rest of this paper is organized as follows. Section 2 systematically presents our sparse solutions for layout and assignment of camera networks. Simulation results are illustrated in Section 3. Discussions and conclusions are given in Section 4. Detailed discussions on the creation of the used anisotropic sensing model are provided in Appendix A.

## 2. Camera Network Deployment

In this section, the sparse representation-based camera network deployment approach is formulated to ensure observation quality and resource consumption reduction.

### 2.1. Observation Quality Measurement

Before performing the deployment, the observation quality of an object point within the sensing field of a camera must be well defined, based on which the overall observation quality of the camera network can be deduced. We construct an anisotropic sensing model, which consists of a non-uniform imaging quality field. In the model, the observation quality is measured by considering four main factors affecting the imaging quality. The factors considered are resolution, defocus, geometric distortion and occlusion, which are represented as *F_r_*, *F_d_*, *F_g_* and *F_o_*, respectively. Observation quality is measured by combining the four components. As the imaging quality will be decreased by the weakening of any one of the components, one camera observation quality of an object point at (*l*, *θ*) is defined as:

(1) $$q\left(l,\theta \right)=\alpha F_{r}\left(l,\theta \right)F_{d}\left(l,\theta \right)F_{g}\left(\theta \right)F_{o}\left(l,\theta \right)$$

where *α* is a normalization parameter and (*l*, *θ*) are the polar coordinates of the object with respect to the camera. This sensing model is systematically described in Appendix A.

Each component describes the distribution feature of sensing quality for the corresponding aspect. Though the observation quality cannot be comprehensively measured by any aspect independently, a reliable measurement is achieved by jointly considering these aspects. With this formulation, the sensing features are reliably described according to the physical imaging process. This measurement reflects the anisotropic characteristic of a camera: directional sensing and non-uniform observation quality field. For a detailed definition of each component in the sensing model, please refer to Appendix A.

When the object is observed by multiple cameras, the synthesized observation quality is the sum of the observation quality of all cameras. We can sum observation quality of different cameras, because multiple views provide more information than one camera. Based on multiview images, we can obtain a high-resolution image/video through super-resolution and solve the occlusion problem for tracking. Meanwhile, the sensing model in Equation (1) takes multiview observation quality into account, where the quality near the boundary of the observation field is penalized exponentially. In addition, the sum operation can be seen as an extension of the *K*-coverage model [16].

Suppose *m* cameras are placed in the scene to be monitored. The *j*th camera has a generalized coordinate, $s_{c}^{j}=\left(p_{c}^{j},o_{c}^{j}\right)$, where $p_{c}^{j}$ and $o_{c}^{j}$ denote the position and the pose of the camera, respectively. Let Ω be the region of the scene. Thus, the overall observation quality of the object located at *p* ∈ Ω is:

(2) $$Q(p)=\overset{m}{\underset{j=1}{\text{∑}}}z_{j}q\;(l(p,s_{c}^{j}),\theta (p,s_{c}^{j}))$$

where $l(p,s_{c}^{j})$ and $\theta \left(p,s_{c}^{j}\right)$ are the conversion functions from the Cartesian coordinates to the polar coordinates and the indicator, *z_j_* ∈ {0, 1}, denotes whether the *j*th camera is selected/activated or not.

### 2.2. Camera Layout

To design a layout algorithm optimizing the resource consumption and observation quality for camera networks, the following aspects should be considered: (a) the lower bound of average observation quality, (b) the uniformity of the observation quality distribution, (c) the minimum resource consumption and (d) regions of different importance.

Let us state the aspects above more intuitively. The lower bound guarantees the minimum observation quality in the scene. Meanwhile, the uniform distribution indicates the equity of observation resource allocation, which avoids both excessively and insufficiently observed areas. Expectation *E*(*Q*) and variance *D*(*Q*) of the observation quality are adopted to measure the distribution of the monitoring resource. Expectation indicates the total amount of observation quality in the scene, while the variance reflects the uniformity. Considering the limited supplies of resource, including hardware cost, energy and bandwidth, a good camera layout should use the least number of cameras to reach both adequate expectation and as low as possible variance of observation quality. In addition, the layout algorithm should have the flexibility of adapting to scenes divided in areas of different importance.

Thus, the optimization goal of camera layout can be mathematically formulated as:

(3) $$\begin{array}{cc} \min & {\left\|z\right\|}_{0}+\mu D\left(Q\right),\\ \text{subject to} & E(Q)\geq E_{0},\\ & Q(p)\geq Q_{0},\;\forall p\in \Omega ,\\ & z_{j}\in \left\{0,1\right\},\;j=1,\cdots ,m \end{array}$$

where ‖*z*‖_0_ denotes the pseudo *ℓ*_0_ norm, which counts the number of non-zero elements in the indicator vector, *z*, with *z* = [*z*_1_, … , *z_m_*]*^T^*, *E*_0_ is the minimum expectation of observation quality, *μ* is a weight coefficient and *Q*_0_ is the minimum observation quality for effective monitoring.

In this formulation, the *ℓ*_0_ minimization is employed as a selector that chooses the smallest subset of cameras from a redundant initial layout. Such a selection provides a near-optimal solution if the initial layout is redundant enough compared with the pure coverage threshold. In other words, the indicator vector, *z*, is very sparse. The minimization of term *D*(*Q*) aims to ensure the uniformity of the observation quality distribution, which can prevent the observation sources from being excessively distributed at the local area. The constraints aim to ensure effective observation coverage on average and on every point.

To solve the optimization (3), we need to compute the expectation, *E*(*Q*), and the variance, *D*(*Q*), of the observation quality with respect to the scene, Ω, being monitored. As the scene, Ω, is continuous in general cases, the expectation and variance can be computed according to:

(4) $$E\left(Q\right)=\frac{1}{\left|\Omega \right|}\iint_{p\in \Omega }Q\left(p\right)/w\left(p\right)\text{d}p$$

(5) $$D\left(Q\right)=E\left\{{\left[Q-E\left(Q\right)\right]}^{2}\right\}=\frac{1}{\left|\Omega \right|}\iint_{p\in \Omega }Q^{2}\left(p\right)/w^{2}\left(p\right)\text{d}p-E^{2}\left(Q\right)$$

respectively, where |Ω| denotes the area of Ω and *w*(*p*) is the importance degree of *p*. The importance degree, *w*(*p*), is introduced to describe critical regions expecting more observation resources. The importance degree is set to *w*(*p*) > 1 and *w*(*p*) = 1 for critical and ordinary regions, respectively.

However, the problem (3) is intractable with the integral form computation of *E*(*Q*) and *D*(*Q*). Firstly, most practical monitoring scenes contain obstacles, and the corresponding boundaries are usually non-convex. That leads to complex discontinuity of *Q*(*p*) in Ω under the visual sensing model. Therefore, integration in the objective function is analytically intractable. Secondly, the observation quality is generally anisotropic in the field of view of the camera, which makes *q*(*l*, *θ*) a quasiconcave (or unimodal) [30] function in its definition domain. Therefore, *Q*(*p*)/*w*(*p*) in Ω is non-concave, because the sum operation does not preserve the quasiconcavity. Thus, the objective function (3) is hard to solve.

To compute *E*(*Q*) and *D*(*Q*), we adopt the numerical integration method. Firstly, *n* points *p_i_* are uniformly sampled from the scene where *i* = 1, ⋯ , *n*. Then, the expectation and variance of observation quality of these sample points are used to approximate the exact *E*(*Q*) and *D*(*Q*). If *n* is large enough, this approximation is accurate enough. To compactly represent the computation of *E*(*Q*) and *D*(*Q*), we define an observation quality matrix, ***B*** ∈ ℝ*^n^*^×^*^m^*, where $B_{\text{ij}}≜q\left(l\left(p_{i},s_{c}^{j}\right),\theta \left(p_{i},s_{c}^{j}\right)\right)$ is the observation quality of point *i* observed by camera *j*. The product, ***B**z*, is a column vector, where the *i*th element is *Q*(*p_i_*). Let ***W*** = diag(1/*w*(*p*_1_), ⋯ , 1/*w*(*p_n_*)) be the weight coefficient matrix. The expectation of observation quality can be approximated by:

(6) $$E\left(Q\right)\approx \frac{1}{n}1^{T}\text{W B}z={\text{b}}^{T}z$$

where 1 is a column vector with *n* ones. With further derivation, the variance is approximated by:

(7) $$D\left(Q\right)\approx \frac{1}{n}\overset{n}{\underset{i=1}{\text{∑}}}{\left(Q\left(p_{i}\right)-E\left(Q\right)\right)}^{2}=\frac{1}{n}{\left\|\text{W B}z-1\left(\frac{1}{n}1^{T}\text{W B}z\right)\right\|}^{2}={\left\|\frac{1}{\sqrt{n}}\left(\text{I}-\frac{1}{n}11^{T}\right)\text{W B}z\right\|}^{2}={\left\|{\text{B}}_{v}z\right\|}^{2}$$

where ***I*** ∈ **R***^n^*^×^*^n^* denotes the unit matrix.

Another difficulty in solving Equation (3) is that the objective function is a non-convex *ℓ*_0_ problem with a Boolean constrain. In order to obtain a convex objective function, we first relax the Boolean constrain to a convex form. Here, we adopt the widely used relaxation method [30], which replaces the Boolean constrain with the linear inequalities as 0 ≤ *z* ≤ 1. Then, we transform the objective to a tractable form. Works [23,24] prove that the real solution can be recovered efficiently by *ℓ*_1_ minimization, as long as the real solution is sparse. In camera layout, *z* is quite sparse, because the number of adopted cameras is very small compared to the redundant initial layout. Therefore, the pseudo *ℓ*_0_ norm can be relaxed to the *ℓ*_1_ norm. As a result, the objective function (3) is relaxed to:

(8) $$\begin{array}{cc} \min & {\left\|z\right\|}_{1}+\mu {\left\|{\text{B}}_{v}z\right\|}^{2},\\ \text{subject to} & {\text{b}}^{T}z\geq E_{0},\\ & \text{W B}z\geq Q_{0}1,\\ & 0\leq z\leq 1 \end{array}$$

according to the sparsity. Compared to the original non-convex objective function, the *ℓ*_0_ item is relaxed to a *ℓ*_1_ norm. *E*(*Q*) and *D*(*Q*) are approximated to two linear calculations. The second constraint was set to ensure the effective observation of the sampled *n* target points of the scene. Moreover, The Boolean constrain is relaxed to a closed interval. As each element of *B* is non-negative, the relaxed constraint lower bounds the total amount of observation quality measured by the original indicator vector.

To obtain the final camera layout, we need to select cameras according to the relaxed non-Boolean indicator, *z*. As the majority elements of *z* are near zero, similar to the way adopted by [31], cameras are selected according to the descending order of coefficients, until the constraints on average and point-wise observation quality in (8) are satisfied. Such a scheme collects the most contributive cameras. Thus, a high quality camera layout is achieved by efficient convex optimization.

### 2.3. Camera Assignment

On the basis of the optimized layout, camera networks can perform a surveillance task of objects appearing in the monitoring scene. To achieve a high efficiency, the cameras’ assignment concerns the following requirements: a) the least number of active cameras, b) observation quality of each observed target and c) a real-time decision.

Camera networks are usually resource (energy and bandwidth)-limited, especially in the wireless environment. The life time of a camera network with limited energy will be greatly decreased if all cameras are activated. The assignment procedure activates the least number of cameras if other constraints are satisfied to reduce the resource consumption. When multiple targets are monitored, different targets generally have different priorities. This is defined as the observation quality requirement for each target. In addition, for surveillance, a real-time decision is indispensable to observe moving targets without interruption.

Suppose m^*^ cameras have been placed in the scene. There are *ñ* points on objects to be observed. Then, observation quality matrix, *B̃* ∈ ℝ*^ñ^*^×^^m^^*^, is obtained, where *B̃_ij_* is the observation quality of point *i* observed by camera *j*. The weighted observation quality of each point must be larger than a given lower bound, *Q̃*_0_, where the weighting matrix is ***W̃*** = diag(*w̃*_1_, ⋯, *w̃_ñ_*). A binary indicator vector, *ψ* = [*ψ*_1_, …, ψ_m__*_]*^T^*, where *ψ_j_* ∈ {0, 1}, is introduced to denote the activation state of each camera. The optimization goal of camera assignment is formulated as:

(9) $$\begin{array}{cc} \min & {\left\|\psi \right\|}_{0},\\ \text{subject to} & \;\tilde{\text{W}}\;\tilde{\text{B}}\psi \geq {\tilde{Q}}_{0}\tilde{1},\\ & \psi_{j}\in \left\{0,1\right\},\;j=1,\cdots ,m* \end{array}$$

where 1̃ is a column vector of *ñ* ones.

The objective function of camera assignment is also an *ℓ*_0_ minimization problem with a Boolean constrain, which is hard to solve in polynomial time. Similar to the procedure used in camera layout, sparse representation is employed to relax this optimization to the *ℓ*_1_ form, because the cameras activated are quite sparse compared to all the placed cameras. Therefore, the objective function is revised to:

(10) $$\begin{array}{cc} \min & {\left\|\psi \right\|}_{1},\\ \text{subject to} & \;\tilde{\text{W}}\;\tilde{\text{B}}\psi \geq {\tilde{Q}}_{0}\tilde{1},\\ & 0\leq \psi \leq 1, \end{array}$$

by means of the sparse property. Cameras are selected into the active subset according to the descending order of coefficients, until the constraints of Equation (9) are satisfied.

The objective functions of camera layout Equation (8) and assignment Equation (10) are both with convex objectives and convex constrains. Thus, they can be solved efficiently via many convex optimization methods, such as interior point, conjugate gradient, *etc.* To solve both optimization problems, we employ CVX, a package for specifying and solving convex programs [32,33].

## 3. Experimental Results

In this section, experiments are designed to demonstrate the effectiveness of the proposed camera deployment algorithm. The camera model adopted in all experiments is with an anisotropic sensing field, which provides an accurate measurement of observation quality and is described in detail in Appendix A. Sensing parameters concerning sensing field and observation quality distribution are derived according to the equipment parameters of the camera. Camera layout and assignment are validated, in turn, on two monitoring scenes. Experimental results of camera layout are analyzed in the following aspects: sparsity, coverage, distribution of observation quality, number of used cameras, optimization time and stability. Then, camera assignment is carried out based on the solution of the layout step. The number of active cameras and the time of assignment decision are used to verify the effectiveness and efficiency, respectively. All the experiments are operated on a computer with a 2.2 GHz CPU and 2 GB RAM.

### 3.1. Configurations of Experiments

**Figure 1.**
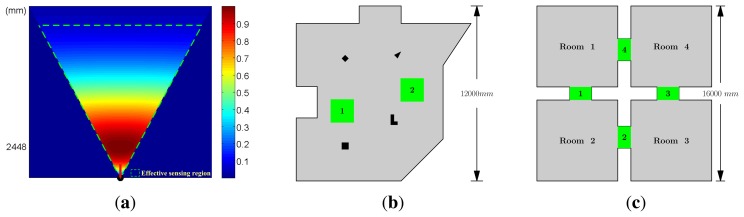
Experimental Parameters. (a) Distribution of observation quality in the sensing field of a selected camera. (b)(c) Two experimental scenes: dark blocks denote the obstacles; green blocks denote the critical regions, which need more observation resources.

Some necessary parameters of device and objective function are set firstly for camera network deployment, as shown in Table 1. All cameras in the network are with the same parameters. The pixel density of the image sensor is 152.17 pixels per square millimeter. The size of the image sensor, *i.e. , D_s_*, in Figure 9, is set to 36 mm. We suppose that at least 2 pixels are needed to observe an object of 5 unit areas effectively. The minimum effective observation quality, *Q*_0_, is set to 0.1. Thus, the parameters of the observation field, such as the field angle and the best object distance, are deduced, as given in Table 1. Sensing field and observation quality distribution are shown in Figure 1a.

Observation quality, *q*(*l*,*θ*), equals to 1 when *l* = *u_best_* and *θ* = 0. Meanwhile, the effective sensing region, determined by setting *q*(*l*, *θ*) ≥ *Q*_0_,is surrounded by a green dashed line.

Two simulated monitoring scenes, which reflect the typical monitor requirements, are designed for experiments. One scene is called the irregular square scene, as shown in Figure 1b. The boundary of this observation scene forms a non-convex polygon. Furthermore, four obstacles, denoted by the dark blocks in Figure 1b, are placed in this scene. This test case carries the complex indoor monitoring scenes. The two green square parts are critical regions. The other is the four rooms scene, which is composed of four rectangular rooms connected by four gangways. This scene simulates the surveillance task with multiple rooms. The shape and size of this scene are shown in Figure 1c. The gangways are defined as the critical regions.

### 3.2. Camera Layout Experiments

The performance of the camera layout algorithm is tested in both designed scenes. To reflect the observation quality of the scene, 900 target points are uniformly sampled. At the initial stage, 1, 000 cameras are randomly placed in the scene. In the construction of the observation matrix, columns with all zero values are deleted, as these vectors represent the ineffective cameras that cannot observe any target points. Thus, they are deleted beforehand to enhance the efficiency of optimization. The minimum expectation observation quality is set to 1.8. Weight coefficient *μ* in the objective function (8) is set to $\sqrt{n}$. The effective coverage is defined as *C* = (|Ω_c_|/|Ω|) x 100%, where the region covered by the observation fields of deployed cameras is denoted by Ω_c_ = {*p*|*p* ∈ Ω,*Q*(*p*) ≥ *Q*_0_}. The coverage is calculated based on the observation data of the target points.

#### 3.2.1. Scenes without Critical Regions

**Figure 2.**
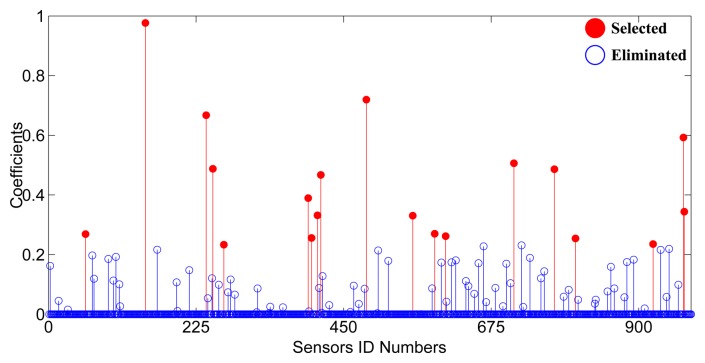
The indicator vector of the layout experiment on the irregular square scene without critical regions. The red dots denote the adopted cameras with large coefficients.

Camera layout is firstly carried out in the irregular square scene, as shown in Figure 1b, without critical regions. The optimization process costs 92.67 seconds to get the coefficients of cameras as shown in Figure 2. There are 962 cameras left after the deletion of ineffective cameras. Only 51 camera coefficients are larger than 0.1. According to the constraints, 19 cameras are chosen as the optimization results. This sparsity can guarantee the effectiveness of *ℓ*_1_ minimization.

**Figure 3.**
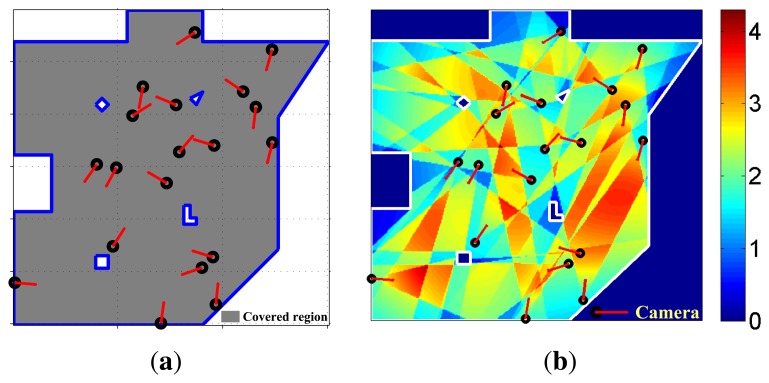
Layout results on the irregular square scene without critical regions. (a) Coverage. (b) Observation quality distribution.

Coverage and observation quality distribution are shown in Figure 3. The coverage of effective observation is 99.74% (the gray part of Figure 3a. Expectation and variance of observation quality are 2.20 and 0.48, respectively. The experimental data shows that almost all parts of the scene are effectively observed.

#### 3.2.2. Scenes with Critical Regions

We then test the camera layout in two scenes with critical regions marked out by green blocks, as shown in Figure 1b,c.

##### The Irregular Square Scene

**Figure 4.**
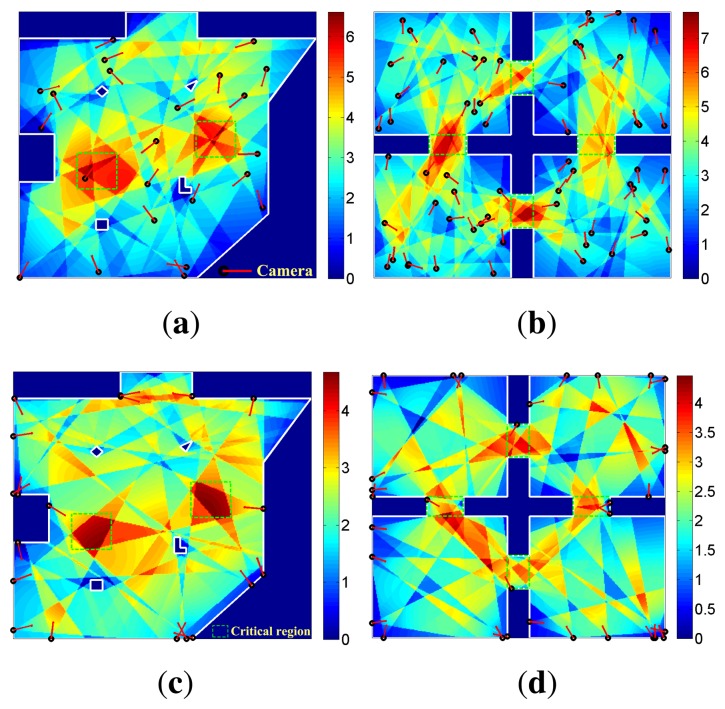
Experimental results of scenes with critical regions. (a) Irregular square scene. (b) Four rooms scene. (c) Boundary restricted layout in irregular square scene. (d) Boundary restricted layout in four rooms scene.

Camera layout is carried out in the scene as shown in Figure 1b, firstly The observation quality distribution is shown in Figure 4a. The observation quality distribution data of this experiment is illustrated in Table 2a. The coverage of the global scene is 96.69%, and both of the two critical regions are completely covered. Since the importance of the whole scene is not equal, variance of observation quality is not given. Experimental data shows that these two critical regions are observed with emphasis compared to common regions. The whole scene is also effectively observed.

##### The Four Rooms Scene

Then, camera layout is carried out in the scene, as shown in Figure 1c. Four gangways marked as green blocks are critical regions. The observation quality distribution is shown in Figure 4b. Observation quality distribution data of this experiment is illustrated in Table 2b. The coverage of Room 4 is 99.88%, and the coverage of all the other regions is 100%. As shown by experimental results, the observation quality distribution of the four rooms can meet the requirement. Moreover, the four gangways are emphatically observed compared to the four rooms.

#### 3.2.3. Boundary-Restricted Layout

In some cases, cameras cannot be placed at all locations of the scene. For example, they can only be placed on the boundary of the monitoring scene. Our layout algorithm can also fit this case effectively.

In this case, cameras are randomly placed on the boundary of the scene at the initial stage. Then, the same operation as the unrestricted layout is executed.

Camera layout on the boundary is carried out, in turn, for the two designed monitoring scenes. Firstly, the camera layout is implemented in the irregular square scene. Observation quality distribution is shown in Figure 4c, and the experimental data is shown in Table 2a. The coverage of the whole scene is 99.59%, and the two critical regions are completely covered.

Then, the boundary-restricted layout is carried out in the four rooms scene. Observation quality distribution and the experimental data are shown in Figure 4d and Table 2b, respectively. The coverage of Room 1 is 99.88%, and the coverage of the other regions is 100%.

As shown by the experimental data, the critical regions are emphatically observed compared to common regions, and the whole scene is also effectively observed though cameras that can only be placed on the boundary. The layouts for different place restrictions verify the robustness of our layout algorithm.

The optimization of these four camera layout experiments in the scenes with critical regions are quite efficient with the sparsity of the layout. Table 3 shows the optimization data of these experiments. As the data show, the comparison between the number of large coefficients and the number of initial effective cameras verifies the sparsity of camera layout, which guarantees the efficiency and accuracy of the optimization algorithm.

#### 3.2.4. Stability of Layout Algorithm

We have performed five camera layout experiments based on different monitoring scenes and layout restrictions. Although the solutions above are obtained from randomly placed cameras, the result of observation quality is stable if the number of initial placed cameras is large enough. The five layout experiments are marked as layout *I* to *V* , as listed in Table 4. In the following, each layout experiment is repeated 50 times under the same parameters. Then, statistical data of objective function values, cameras number, coverage and observation quality distribution for different layout experiments are obtained.

##### Objective Function Values

**Figure 5.**
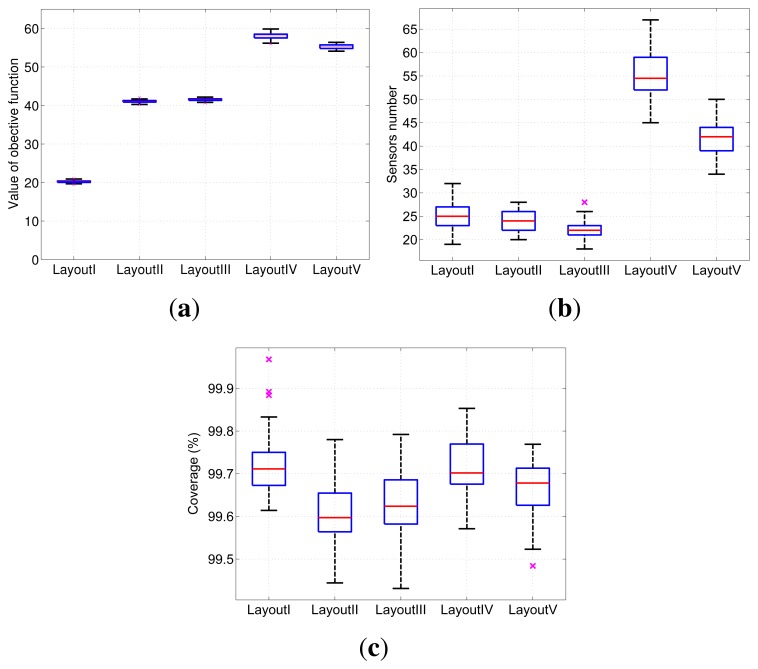
Statistical data of the deployment performance of the layout experiments (a) The objective value. (b) The number of adopted cameras. (c) Effective coverage.

The stability of the values of objective function (8) reflects the convergence of the layout optimization algorithm. Boxplots of these values for the five layout experiments are given in Figure 5a. As shown in the figure, the values of objective functions (8) for the five experiments are quite stable. This result proves that there exists a large number of high quality solutions with similar objective values among the optimal solution, and they can be effectively obtained by the proposed algorithm with random initial camera layout.

##### Number of Adopted Cameras

The number of adopted cameras placed in the scene reflects the layout cost of camera networks. The boxplots of the number of adopted cameras in the five layout experiments are shown in Figure 5b. We can see that the length of every box is very short compared to the value of the median data. The stability of cameras number is reflected.

##### Effective Coverage

Effective coverage of the scene is an important aspect of QoS. Blind areas should be as small as possible in the monitoring scene. Statistical data of the coverage of five layout experiments are shown in Figure 5c. Because the coverage of all critical regions is 100%, only the global coverage data is given. It can be seen in this diagram that coverage of each layout experiment is greater than 99%. Sufficient, effective coverage is guaranteed.

##### Distribution of Observation Quality

**Figure 6.**
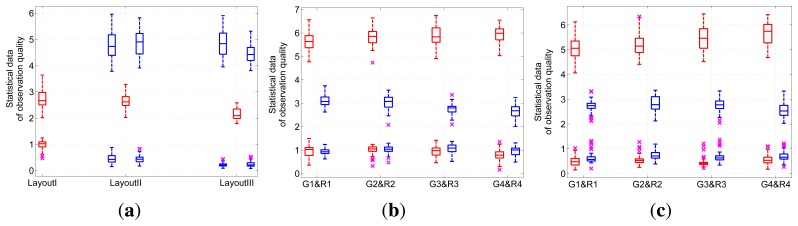
Statistical data of the observation quality distribution of five layout experiments. Blue boxes are the statistical data of critical regions. Red boxes reflect the statistical data of common regions. An upper box and lower box with the same abscissa denote the expectation and variance of the observation quality, respectively. (a) Layout *I*–Layout *III*. (b) Layout *IV*. (c) Layout *V*. In the figures, G stands for gangway and R for room.

The first three layout experiments in the irregular square scene are tested, firstly. Figure 6a shows the statistical data of the observation quality distribution of these three layout experiments. As Layout *I* shows, the expectation and variance are comparatively concentrated. Boxplots of Layout *II* and Layout *III* also reflect the stability of the deployment solutions.

Then, Figure 6b shows the statistical data of the observation quality distribution of the camera layout experiment in the four rooms scene without placement restriction. Statistical data of the observation quality distribution in gangways and rooms show the stable quality of layout solutions.

Finally, for experiments of boundary-placed camera layout in the four rooms scene, Figure 6c shows the statistical data of the observation quality distribution. Similar to the previous experiments, the deployment solutions of our approach are stable at an acceptable interval.

### 3.3. Comparison to BIP-Based Deployment

In addition, in order to verify the superiority of our optimization objective function, it is compared to binary integer programming (BIP)-modeled deployment based on binary sensing nodes. A comparison experiment is made between our formulation and [8] in a scene three-times larger than the scene shown in Figure 1b. In this experiment, M1 denotes the deployment under the binary sensing model with the BIP objective function, which is designed in [8]. M2 denotes the deployment under our nonuniform sensing model and sparse relaxation-based method. The uniformity restriction on the observation quality distribution, *i.e.*, the variance term, is added to our objective function, but ignored in M1. The two experiments are with the same observation requirements. For M1, we segment the field of view by setting a threshold on observation quality and restrict the observation quality of any part of the scene to not less than one. For M2, we restrict the observation quality of any part of the scene to no less than this threshold.

**Figure 7.**
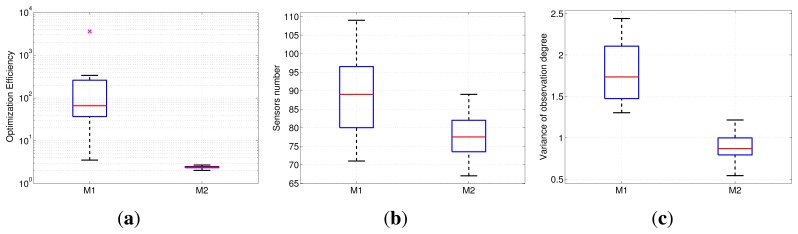
Comparison between the binary integer programming (BIP) objective function with the binary model and our objective function with the nonuniform model. (a) The optimization time for the two objective functions. (b) The number of deployed cameras. (c) The variance of the observation quality distribution.

To compare the optimization efficiency between both models, the deployment is firstly operated on a small scale problem, which is with 15 × 15 target points and 200 initial cameras. As shown by Figure 7(a), the optimization time of M2 stabilizes at two seconds, while the branch and bound-based M1 model is quite unstable (MATLAB bintprog function, which solves the BIP problem via the branch and bound method). Thus, the optimization efficiency performance of M2 is much better than the M1 model.

Then, in order to compare the deployment quality, a problem with large scale, 30 x 30 target points and 900 initial cameras is tested. The M1 model fails for this scale problem, which cannot output the solution within one hour using the bintprog function. Thus, we adopt the *ℓ*_1_ relaxation.

The experimental data are illustrated by boxplots, as shown in Figure 7, where each box is a collection of observation data with 20-times repeated experiments under the same parameter settings. In the experiment, each pair of results of M1 and M2 are obtained based on the same initial layout. Our optimization model has less requirement of cameras. In addition, our solution can achieve smaller variance of observation quality than the M1 result, which means a more balanced observation resource distribution in the scene. This result verifies that our approach can obtain the layout with less cameras and inhibit the excessive observation of somewhere in an equally important region, which is better than the M1 model by means of our nonuniform sensing model and a reasonably objective function.

### 3.4. Camera Assignment Experiments

In this section, we test the effectiveness of camera assignment based on previous layout results. Different numbers of objects are randomly placed in the monitoring scenes, and each object is given a threshold of observation quality.

**Figure 8.**
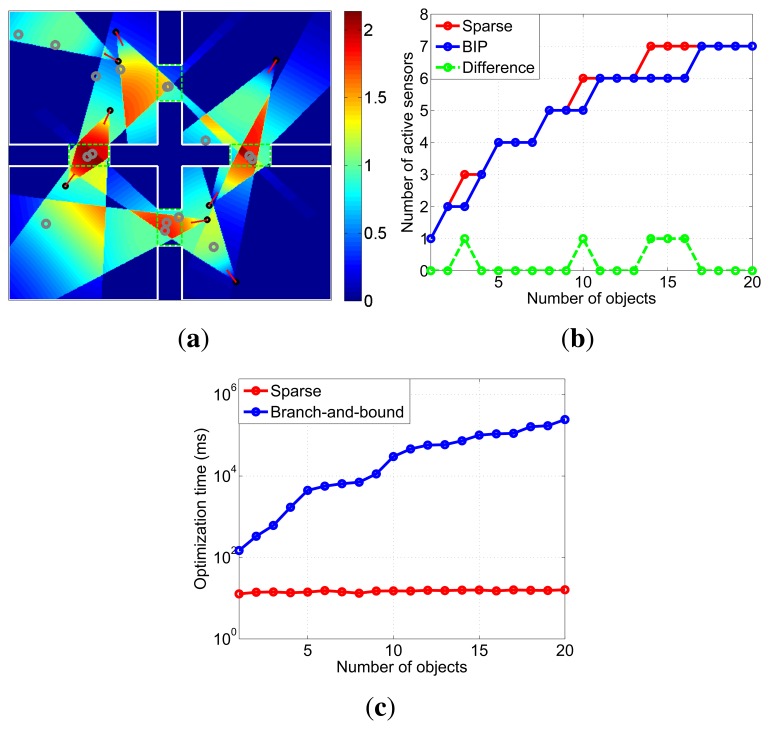
Camera assignment results based on the four rooms scene. (a) Observation effect of camera assignment. The gray rings are the objects. (b) Active camera number obtained by two methods. (c) Optimization time of two methods.

**Figure 9.**
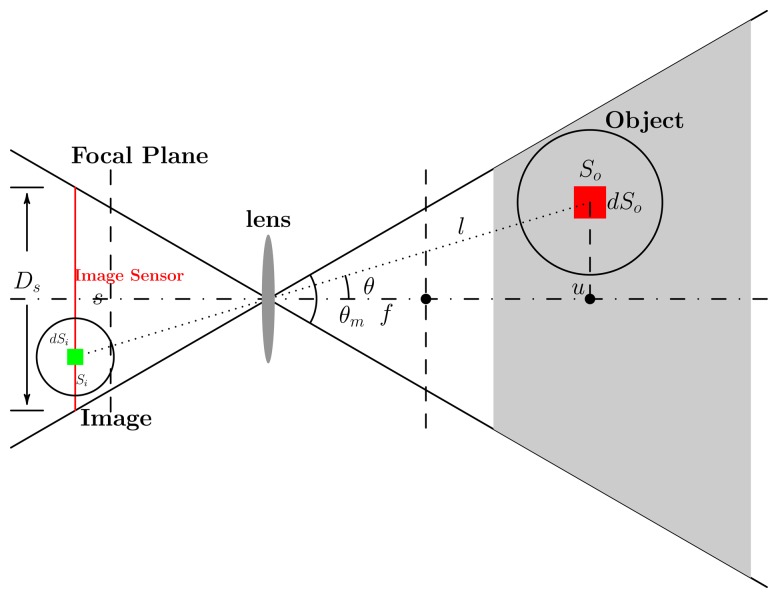
The 2D imaging model of a camera. The gray part is the effective sensing field. The area element, d*S_i_*, on image is the projection of the area element, d*S_o_*, on the object.

Camera assignment is tested based on the four rooms scene. Observation quality thresholds of objects in the critical region are set to 0.5 and 1, respectively. The assignment result is shown in Figure 8. There are 16 objects in the scene, and eight of them are critical objects. In order to observe these objects effectively, eight cameras are activated. The assignment decision time are 15*ms*.

As shown in Figure 8, each object in the monitoring scene is effectively observed, satisfying their observation quality thresholds. The effectiveness of the proposed assignment strategy is validated.

A real-time decision is indispensable to observe targets without interruption in practical application, such as object tracking. If the assignment objective function (9) is directly solved by BIP without any convex relaxation strategy, the decision time becomes unacceptable when the number of cameras and objects are large. However, our approach can obtain the active cameras within a few milliseconds by using the sparsity of assignment. Then, comparisons of optimization performance and efficiency between BIP and the sparse approach are made, respectively. We adopt MATLAB function *bintprog* as the optimization function of BIP. The number of active cameras obtained by BIP is viewed as the optimal solution, *i.e.*, the least number of active cameras. Based on the node layout, as in Figure 4b, the comparative data of objects numbered from 1 to 20 are shown in Figure 8.

As shown in Figure 8b, solutions of two methods with different numbers of objects are almost the same. However, with the growing number of objects, the optimization time of BIP increases rapidly, as shown in Figure 8c. Thus, BIP cannot be used in real-time multi-object surveillance systems. In contrast, no matter how many objects in the scene, our approach can give exact assignment decisions in 14–16 ms. Therefore, sparse representation is quite valuable for real-time camera assignment.

## 4. Discussions and Conclusions

Firstly, the proposed sparsity-induced convex relaxation is quite valuable, because this approach can achieve both high quality and efficiency for large-scale deployment. Though BIP-based deployment can be solved by employing the branch and bound method, it is not efficient enough, as it can only be applied to small-scale problems and cannot handle a large number of initial cameras. With a limited number of initial cameras, this has no guarantee to the quality of the resulting deployment.

Secondly, the good stability of optimization solutions verifies the practicality of the proposed deployment approach. Based on a sufficiently redundant initial deployment, our method can obtain solutions with similar qualities in statistical experiments. There lacks a theoretical basis about whether the solution is the optimal solution or a near-optimal solution in this paper and existing works, which is quite important for camera deployment. However, sufficiently redundant initial deployment does cover an acceptable solution for practical usage.

In this paper, we propose an efficient observation quality-optimized camera deployment algorithm based on a newly designed sensing model. On the basis of the sensing model, the camera deployment algorithm efficiently deals with the camera layout and assignment problems. With a constructed sparse representation, the original non-convex camera deployment problem is tackled by convex optimization with a high quality solution. The high efficiency and stability of the proposed approach are confirmed through comprehensive experiments.

In addition, the sparsity of deployment is independent of the concrete sensing model of the camera, which means that the proposed sparse formulation can be extended to a wide variety of applications, including even other types of sensor networks. Next, we plan to extend the study to the collaboration strategies of camera networks for visual information acquisition in surveillance systems.

## Figures and Tables

**Figure 10.**
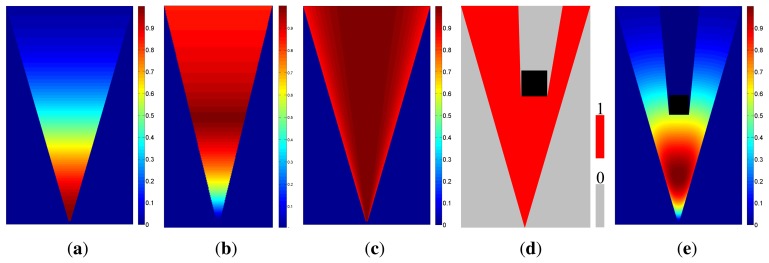
The distribution in the sensing field of the four considered components. (a) Resolution component. (b) Defocus component. (c) Geometric distortion component. (d) Occlusion effect caused by the obstacle. (e) Distribution of observation quality in the sensing field with obstacles.

**Figure 11.**
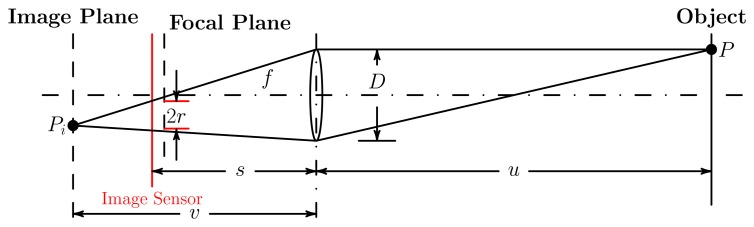
Principle diagram of the defocus effect.

**Table 1.** Sensing parameters of adopted cameras.

Focal Length (*f*)	50 *mm*	F-number (*f*/*D*)	1.8
*κ*	0.01	*d*_max_	*D_s_*/2
*r_m_*	*D*/8	*F_r_*_,min_/*F_d_*_,min_/*F_g_*_,min_	0.05

*θ_m_*	0.69 *rad*	*u_best_*	2, 448 *mm*

**Table 2.** Observation quality distribution data of scenes with critical regions.

(a) The Irregular Square Scene				

		Global	Region 1	Region 2
Free	*E*(*Q*)	2.88	5.50	5.18
	*D*(*Q*)	/	0.17	0.32

Boundary	*E*(*Q*)	2.36	4.04	4.09
	*D*(*Q*)	/	0.34	0.35

(b) The Four Rooms Scene (RM—room, GW—gangway.)									

		RM 1	RM 2	RM 3	RM 4	GW 1	GW 2	GW 3	GW 4
Free	*E*(*Q*)	3.19	3.31	2.97	2.88	5.97	5.55	5.09	4.37
	*D*(*Q*)	0.96	1.31	1.08	1.25	1.24	1.93	0.32	1.76

Boundary	*E*(*Q*)	2.04	1.96	1.97	1.96	2.73	2.73	2.78	3.13
	*D*(*Q*)	0.55	0.60	0.40	0.45	0.57	0.40	0.75	0.55

**Table 3.** Experimental data of layout optimization in two scenes with critical regions.

	Irregular Square Scene		Four Rooms Scene	

	Free	Boundary	Free	Boundary
Effective Cameras	957	825	943	778
Large Coefficients (> 0.1)	33	29	66	58
Adopted Cameras	23	19	63	32
Optimization Time (s)	120.49	126.29	143.88	139.63

**Table 4.** List of the sequence number for cameras layouts.

Irregular square scene without a critical region	Layout *I*
Irregular square scene with critical regions	Layout *II*
Boundary-restricted irregular square scene	Layout *III*
Four rooms scene	Layout *IV*
Boundary-restricted four rooms scene	Layout *V*

## A. Appendix A. Camera Modeling

A novel anisotropic sensing model of a camera is proposed to measure the observation quality. The sensing features of cameras are different from other types of sensors. A camera can only observe objects within a certain viewing angle range and depth. In addition, the distribution of observation quality in the sensing field is nonuniform, *i.e.*, anisotropic sensing.

As the classical pinhole model shows, the image resolution of a target depends on its distance to the camera and is measured by the ratio between the projection area on the focal plane of the target and the area of one pixel. Moreover, the defocus effect becomes non-negligible when the object is out of focus and reduces the sharpness of an image [34,35]. Furthermore, imaging through an optical lens does not strictly comply with the pinhole model for the radial distortion. The projection of a straight line in the scene cannot remain straight in the image, especially at the edge of the view. In addition, the image sensing can easily be occluded by obstacles.

Therefore, resolution, defocus, geometric distortion and occlusion are four main factors affecting imaging quality. All of them are crucial to feature extraction and image analysis. Though other factors, such as noise and exposure value, can also affect imaging quality, they can be adjusted into an acceptable range beforehand by modifying the parameters of cameras and scenes. In the following, four functions are designed to measure the observation quality determined by these factors, respectively. The anisotropic sensing model for cameras is derived by combining these functions.

The imaging principle of the camera is based on the pinhole model. Its 2D model is shown in Figure 9. The image, *S_i_*, of object *S_o_* is projected on the image sensor through the lens. Suppose the length of the image sensor is *D_s_*. The distance between the image sensor and the lens is *s*. The field angle, *θ_m_*, is calculated as 2 arctan(*D_s_*/2*s*). The object can be projected onto the image sensor only when *θ* ≤ *θ_m_*/2.

### A.1. Resolution

Resolution is the most intuitive factor affecting imaging quality. An image with a higher resolution can provide more details of the object. The image resolution of the object is directly determined by the area of the image and the pixel density of an image sensor. As shown in Figure 9, let d*s_o_* be the area of a small part on the surface of an object and d*s_i_* the area of its image. The proportion between d*s_o_* and d*s_i_* is:

(11) $$\begin{array}{cc} \frac{\text{d}s_{o}}{\text{d}s_{i}}=\frac{u^{2}}{s^{2}}, & \text{with}\;u=l\cos\theta . \end{array}$$

The observation quality, *F_r_*, with respect to resolution is defined as:

(12) $$F_{r}\left(l,\theta \right)=\exp\left(-\frac{\text{d}s_{o}/\text{d}s_{i}}{\sigma_{r}}\right)=\exp\left(-\frac{l^{2}\cos^{2}\theta }{\sigma_{r}s^{2}}\right),$$

where *σ_r_* is a parameter that controls the decay rate of *F_r_*.

The distribution of the resolution component in the sensing field is shown in Figure 10a. It shows the decay process of the resolution component with increasing object distance, *u*.

### A.2. Defocus

The defocus phenomenon reduces the sharpness of an image. The distance between the image plane and the image sensor plane cannot be ignored when the object is out of focus. Thus, the image of the object on the image sensor is blurred. The principle of defocus is shown in Figure 11.

As shown in Figure 11, the distance between the image sensor (e.g., a CCD or CMOSarray) and the focused image plane, (|*v* — *s*|), is non-negligible when the object is out of focus. Thus, a point, *P*, of the object is imaged as a blur circle of radius, *r*, *i.e.*, the defocus. The calculation of *r* is given by [34,35] as:

(13) $$r=\frac{\text{sD}}{2}\left|\frac{1}{f}-\frac{1}{u}-\frac{1}{s}\right|,$$

where *D* is the diameter of the lens.

The defocused image is composed of many blur circles. Thus, the visual effect of the defocused image is not good, because of the low sharpness. Defocus effect becomes more serious with the increase of *r*. The observation quality related to the defocus aspect is defined as:

(14) $$F_{d}\left(l,\theta \right)=\exp\left(-\frac{r}{\sigma_{d}}\right)=\exp\left(-\frac{\text{sD}}{2\sigma_{d}}\left|\frac{1}{f}-\frac{1}{l\cos\theta }-\frac{1}{s}\right|\right),$$

where *σ_d_* is a parameter that controls the change rate of *F_d_*.

The optimal focus distance is calculated as *u*_opt_ = *fs*/|*s* – *f*| by setting *r* = 0. The distribution of *F_d_* in the sensing field with a given *u*_opt_ is shown in Figure 10b. The range, including *u*_opt_, with high a defocus component value, is seen as the depth of field of the camera. In experiments, because we do not know the positions of objects and cameras before the layout stage, the *u*_opt_ is set to infinity, that is, *f* = *s*.

### A. 3. Geometric Distortion

Nonlinear geometric distortion leads to deviations from linear projection. That is to say, a projection of a straight line in the scene through an optical lens cannot remain a straight line in the image.

The radial distortion model [36], which is the most commonly encountered geometric distortion, provides a one parameter formulation. Suppose the coordinate of a point in an undistorted image is (*x*, *y*); the corresponding coordinate in the distorted image is (*x’*,*y’*). The approximate transform is given as:

(15) $$\begin{array}{cc} x^{\prime }=x+\kappa x\left(x^{2}+y^{2}\right), & y^{\prime }=y+ \end{array}\kappa y\left(x^{2}+y^{2}\right),$$

where *κ* is a parameter decided by the lens.

Let *d_r_*, which equals *s* tan *θ* in a 2D sensing model, be the distance on the image between a pixel and the image center. The distorted deviation, $d_{r}^{\prime }=d_{r}\left(1+\kappa {d_{r}}^{2}\right)$. Observation quality of geometric distortion aspect is defined as:

(16) $$F_{g}\left(\theta \right)=\exp\left(-\frac{\Delta d_{r}/D_{s}}{\sigma_{g}}\right)=\exp\left(-\frac{\left|\kappa \right|s^{3}\tan^{3}\theta }{D_{s}\sigma_{g}}\right),$$

where $\Delta d_{r}=\left|d_{r}-d_{r}^{\prime }\right|$. The parameter, *σ_g_*, controls the decrease rate of *F_g_*. The distribution of the geometric distortion component in a sensing field is shown in Figure 10c. It shows the decreasing process of the geometric distortion component with increasing angle of view, *θ*.

In computer vision, it has been well known that camera radial distortion can also be compensated for by a standard calibration procedure [37]. However, as calibration is generally a resampling method based on the distorted image, it cannot make a remedy for the loss of observation quality, due to the corruption of the uniform sampling scheme caused by nonlinear geometric distortions. In addition, calibration also cannot compensate for the lost parts near the four borders of the view. Thus, distortion is an essential factor in the observation quality model.

### A.4. Occlusion

Visual sensing is easily occluded by obstacles in the sensing field of cameras. If there is no obstacle between the object and the camera, the observation quality is determined by the first three components; however, the observation quality becomes zero, as long as the line of sight is occluded by the obstacle. Thus, the occlusion component, *F_o_*, is defined as:

(17) $$F_{o}\left(l,\theta \right)=\{\begin{array}{ll} 1, & \text{Unoccluded},\\ 0, & \text{Occluded}. \end{array}$$

The distribution of the occlusion component in the sensing field is shown in Figure 10d.

### A. 5. Decaying Parameters

In order to obtain the parameters, *σ_r_*, *σ_d_*, *σ_g_*, for the first three observation aspects, the measurement of each component is set with a threshold, while the quality of the physical imaging process satisfies the minimum requirements, which are:

(18) $${F_{h}\left(l,\theta \right)|}_{\text{cond}}=F_{h,\min,}$$

where the subscript, *h*, denotes one of the three aspects and cond denotes the critical condition of the corresponding aspect. For the resolution component, *F_h_* denotes *F_r_*. The critical condition is the imaging resolution of an object with the unit area, *R*_min_, which is d*s_o_*/d*s_i_* = 1/(*s_p_R*_min_), where *s_p_* is the area of one pixel. For the defocus component, *F_h_* denotes *F_d_*, which equals *F_d_*_,min_, as long as the radius of the blur circle, *r*, equals *r_m_*. Finally, for the geometrical distortion component, *F_h_* denotes *F_g_*. The distortion achieves the the critical condition when Δ*d_r_* = *d*_max_.

Through considering these four components comprehensively, the observation quality is measured as *q*(*l*, *θ*) = *αF_r_*(*l*, *θ*)*F_d_*(*l*, *θ*)*F_g_*(*θ*)*F_o_*(*l*, *θ*), which is described in Section 2.1. The distribution of observation quality in the sensing field, which contains a square obstacle, is shown in Figure 10e.
